# Randomized pharmacokinetic evaluation of different rifabutin doses in African HIV- infected tuberculosis patients on lopinavir/ritonavir-based antiretroviral therapy

**DOI:** 10.1186/2050-6511-15-61

**Published:** 2014-11-19

**Authors:** Suhashni Naiker, Cathy Connolly, Lubbe Wiesner, Tracey Kellerman, Tarylee Reddy, Anthony Harries, Helen McIlleron, Christian Lienhardt, Alexander Pym

**Affiliations:** TB Research Unit, Medical Research Council, Durban, South Africa; Biostatistics Unit, Medical Research Council, Durban, South Africa; Division of Clinical Pharmacology, Department of Medicine, University of Cape Town, Cape Town, South Africa; International Union Against Tuberculosis and Lung Disease, Paris, France; WHO STOP Tuberculosis Programme, Geneva, Switzerland; KwaZulu-Natal Research Institute for Tuberculosis and HIV (K-RITH), University of KwaZulu-Natal, Durban, South Africa

**Keywords:** Rifabutin, Pharmacokinetics, Lopinavir, Tuberculosis, HIV, DDI, Randomized, Clinical trial, Neutropenia, Uveitis

## Abstract

**Background:**

Pharmacokinetic interactions between rifampicin and protease inhibitors (PIs) complicate the management of HIV-associated tuberculosis. Rifabutin is an alternative rifamycin, for patients requiring PIs. Recently some international guidelines have recommended a higher dose of rifabutin (150 mg daily) in combination with boosted lopinavir (LPV/r), than the previous dose of rifabutin (150 mg three times weekly {tiw}). But there are limited pharmacokinetic data evaluating the higher dose of rifabutin in combination with LPV/r. Sub-optimal dosing can lead to acquired rifamycin resistance (ARR). The plasma concentration of 25-O-desacetylrifabutin (d-RBT), the metabolite of rifabutin, increases in the presence of PIs and may lead to toxicity.

**Methods and results:**

Sixteen patients with TB-HIV co-infection received rifabutin 300 mg QD in combination with tuberculosis chemotherapy (initially pyrazinamide, isoniazid and ethambutol then only isoniazid), and were then randomized to receive isoniazid and LPV/r based ART with rifabutin 150 mg tiw or rifabutin 150 mg daily. The rifabutin dose with ART was switched after 1 month. Serial rifabutin and d-RBT concentrations were measured after 4 weeks of each treatment. The median AUC_0–48_ and Cmax of rifabutin in patients taking 150 mg rifabutin tiw was significantly reduced compared to the other treatment arms. Geometric mean ratio (90% CI) for AUC_0–48_ and Cmax was 0.6 (0.5-0.7) and 0.5 (0.4-0.6) for RBT 150 mg tiw compared with RBT 300 mg and 0.4 (0.4-0.4) and 0.5 (0.5-0.6) for RBT 150 mg tiw compared with 150 mg daily. 86% of patients on the tiw rifabutin arm had an AUC0-24 < 4.5 μg.h/mL, which has previously been associated with acquired rifamycin resistance (ARR). Plasma d-RBT concentrations increased 5-fold with tiw rifabutin dosing and 15-fold with daily doses of rifabutin. Rifabutin was well tolerated at all doses and there were no grade 4 laboratory toxicities. One case of uveitis (grade 4), occurred in a patient taking rifabutin 300 mg daily prior to starting ART, and grade 3 neutropenia (asymptomatic) was reported in 4 patients. These events were not associated with increases in rifabutin or metabolite concentrations.

**Conclusions:**

A daily 150 mg dose of rifabutin in combination with LPV/r safely maintained rifabutin plasma concentrations in line with those shown to prevent ARR.

**Trial registration:**

ClinicalTrials.gov Identifier: NCT00640887

## Background

Treating HIV associated tuberculosis remains a formidable challenge. In 2014, 13% of the 9 million incident cases of tuberculosis, and 25% of deaths from tuberculosis were in HIV-infected patients [1]. Combining efavirenz-based first-line antiretroviral therapy (ART) with rifampicin based tuberculosis chemotherapy significantly reduces mortality in these patients [2–4] and is safe and efficacious. However, as public sector ART expands in developing countries, an increasing number of patients are developing virological failure and require second-line ART with protease inhibitors [5, 6]. Combining rifampicin and protease inhibitor-based second-line ART is problematic as rifampicin significantly reduces the bioavailability and increases the clearance of protease inhibitors by accelerating their metabolism via induction of cytochrome 3A4 (CYP3A4) enzymes. Increasing the dose of the protease inhibitor or co-administering higher doses of a CYP3A4 inhibitor to ameliorate this adverse drug-drug interaction have been thwarted by hepatotoxicity and other problems with tolerability [7, 8].

Rifabutin, a less potent inducer of CYP3A4 [9, 10], is recommended at 300 mg daily as prophylaxis and treatment of *Mycobacterium avium* complex (MAC) and for the treatment of drug susceptible tuberculosis. Plasma concentrations of rifabutin are increased in the presence of protease inhibitors [11] therefore dose adjustments are recommended when rifabutin is combined with a protease inhibitor. Some recent guidelines recommend dosing rifabutin 150 mg daily (QD) in combination with a ritonavir-boosted protease inhibitor [12], but others still recommend 150 mg three time weekly (tiw) [13]. These differences in guidelines are due to the limited pharmacokinetic studies comparing the 2 dosing regimens of rifabutin and persisting concerns about the tolerability and toxicity of using higher doses of rifabutin [14]. Previous reports suggested that less frequent dosing at 150 mg in HIV-positive tuberculosis patients can result in inadequate rifamycin concentrations [15, 16], relapse [17] and acquired rifamycin resistance (ARR) [18]. Patients with rifabutin AUC_0–24_ < 4.5 μg.h/mL were identified as at the highest risk of ARR. The optimum pharmacokinetic parameter associated with treatment efficacy is unknown.

Elimination of rifabutin is primarily by metabolism via various routes, with deacetylation to d-RBT considered the most important. The d-RBT metabolite is known to have antibacterial activity [19] but may also contribute to toxicity, and is thought to be metabolized further in the liver by CYP 3A4. The present study was therefore undertaken to compare the bioavailability of rifabutin and d-RBT after two different dosing regimens of rifabutin (150 mg tiw and 150 mg daily) in combination with ritonavir boosted lopinavir (LPV/r), the protease inhibitor most commonly used to treat HIV infection in South Africa.

## Methods

### Study design

**Figure 1:**
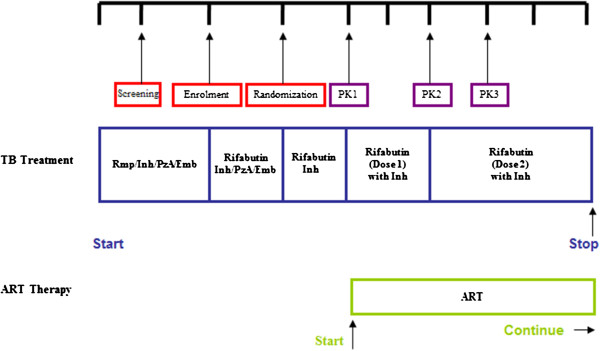
**Diagram showing the timings of clinical trial visits and study regimens to tuberculosis (TB) treatment.** Patients were screened after 5 weeks of standard TB chemotherapy administered as a fixed dose combination (Rmp – rifampicin, Inh – isoniazid, Pza – pyrazinamide, Emb – ethambutol). If patients met all eligibility criteria they were enrolled after 6 weeks of TB chemotherapy and switched to rifabutin 300 mg daily in place of rifampicin. At the end of the intensive phase (8 weeks of TB treatment) they continued with rifabutin 300 mg daily and isoniazid 300 mg daily. This was followed by the first pharmacokinetic visit (PK1) at which the bioavailability of rifabutin in the absence of LPV/r was assessed. The patients then initiated antiretroviral therapy (ART) and altered their dose of rifabutin based on the randomization to either 150 mg tiw of 150 mg daily. After a month of ART a second pharmacokinetic evaluation (PK2) was completed. Patients then switched doses of rifabutin from 150 mg tiw to daily, or vice versa, and after a further month of treatment a third pharmacokinetic evaluation was completed (PK3). Patients then continued with rifabutin at the dose they were on at PK3, in combination with ART and isoniazid until a total of 24 weeks of TB treatment had been completed. Patients continued ART after stopping TB treatment.

An open-label, randomized, three-period, crossover drug interaction study was undertaken to investigate the pharmacokinetics of rifabutin with and without PI-based ART (Figure 1). The secondary objective was to assess the tolerability and safety of rifabutin and LPV/r. The Biomedical Research Ethics Committees of the Universities of Kwa-Zulu Natal and Cape Town, and the Ethics Committee of the International Union against Tuberculosis and Lung Disease (Paris) and the South African Medicines Control Council approved the study. The trial registration number was NCT00640887 (https://clinicaltrials.gov/).

### Recruitment

Patients were recruited from local tuberculosis clinics in Kwa-Zulu Natal, South Africa. The study ran from February 2009 until October 2010. All patients provided written informed consent. Eligibility requirements were a diagnosis of pulmonary tuberculosis confirmed by microscopy or culture, HIV infection with CD4 lymphocyte count ≥50 and ≤200 cells/mm^3^, weight ≥50 kg or a BMI ≥18, a Karnofsky score Q ≥80% and no grade 3 or 4 clinical or laboratory findings according to DAIDS tables [20]. The CD4 restrictions for this study were a reflection of the South African guidelines for the initiation of ART in TB patients at the time the study was conducted [21]. Patients with CD4 counts below 50 were recommended to initiate therapy immediately whereas those with CD4 count between 50 and 200 were initiated at 2 months of TB therapy.

Only patients who completed and adhered to 6 weeks of standard intensive phase chemotherapy and had not received ART therapy in the preceding three months were enrolled. Patients with a previous tuberculosis episode within three years prior to the current episode, a history of prior treatment for MDR tuberculosis, concomitant opportunistic infection requiring additional anti-microbial treatment, a formal contraindication to any trial medication, diabetes mellitus requiring treatment, recreational drug or alcohol abuse, mental illness, total neutrophil count <1200 cells/L, hemoglobin <6.8 g/dL, or liver function tests > grade 2, pregnancy or lactating women were excluded.

### Treatments under study

At enrollment, six weeks after starting standard tuberculosis chemotherapy, rifampicin was switched to rifabutin 300 mg daily (Figure 1). After two weeks of rifabutin, pyrazinamide and ethambutol were stopped and patients continued with daily doses of rifabutin 300 mg in combination with isoniazid 300 mg. After two more weeks, the first pharmacokinetic evaluation (PK1) was carried out and patients were randomized to one of two different rifabutin dose sequences together with daily doses of isoniazid and ART comprising LPV/r (400/100 mg) plus lamivudine (150 mg bd) and stavudine (30 mg bd). Half the patients received rifabutin 150 mg tiw for 4 weeks before being switched to rifabutin 150 mg daily after a second pharmacokinetic evaluation (PK2). A third pharmacokinetic evaluation (PK3) took place after 4 weeks and they remained on this dose of rifabutin until completion of tuberculosis treatment. Half the patients received the two rifabutin doses in a reverse sequence.Physical examinations and laboratory investigations were done at screening, after 1 month of rifabutin (trial day 28 – PK1), after 1 month of ART and rifabutin (trial day 56 – PK2), after 2 months of ART and rifabutin (trial day 84 – PK2) and 2 weeks before the end of TB treatment (trial day 112) as shown in Figure 1. Upon completion of the trial, patients were referred to local antiretroviral clinics for further management. Pfizer (South Africa) supplied the rifabutin (Mycobutin^®^) 150 mg capsules and the new film-coated tablet formulation of LPV/r, Aluvia^®^ was purchased from Abbott Laboratories (USA).

### Sample size

Based on the AUC_0–24_ for rifabutin determined in previous studies, it was estimated that a sample size of 12 participants had a power of 80% to detect a 20% difference between the mean AUC_0–24_ for rifabutin with and without ART. The sample size was calculated on the assumption that 16 enrolled participants would result in a minimum of 12 evaluable subjects. The additional 4 patients in each arm were recruited as it was thought that there may be drop out of patients before completing 3 full pharmacokinetic visits.

### Pharmacokinetic sampling

All patients were admitted before each pharmacokinetic occasion and were fasted from midnight. A standard hospital breakfast (oats with 2 slices of toast and tea) was served 2 h after drug ingestion. Blood draws were done at 0, 2, 3, 4, 5, 6, 8, 12, 24 and 48 h after drug ingestion. The samples were placed on ice immediately and centrifuged at 3000 rpm at 4°C for 10 minutes. Separated plasma was stored immediately at −70°C until batch analysis.

### Drug analyses

Rifabutin and d-RBT were analyzed with a validated LC/MS/MS assay [14]. Rifaximin was used as internal standard at a concentration of 100 ng/ml. Gradient chromatography was performed on a Phenomenex, Luna 5 μm PFP (2), 100 A, 50 mm × 2 mm analytical column, using acetonitrile and 0.1% formic acid as mobile phase, and a flow rate of 500 μl/min. An AB Sciex API 3200 mass spectrometer monitored protonated ions at m/z 847.4 to the product ions at m/z 95.1 for rifabutin, at m/z 805.4 to the product ions at m/z 95.1 for d-RBT, and at m/z 786.3 to the product ions m/z 151.1 for rifaximin. Rifabutin and d-RBT accuracies were between 99.1% and 109.0% during inter-batch validation. The co-efficient of variation during inter-batch validation was less than 9.2%. The calibration range for rifabutin was between 3.91 ng/ml and 1000 ng/ml, and for d-RBT between 0.780 ng/ml and 200 ng/ml. The intra- and inter-batch accuracy statistics of the rifabutin and d-RBT assay validation were between 93.3% and 111.5%, and between 99.1% and 109%. The co-efficient of variation was less than 13.8%.

Plasma lopinavir concentrations were quantified by a validated LCMS/MS method previously described by Chi et al. [22]. The calibration curve was linear over the range from 0.05 to 20 mg/L. Samples with a concentration of >20 mg/L, were diluted and re-analyzed. Any sample below the LLQ was reported as 0.5 X LLQ for analysis. The intra- and inter-batch accuracy statistics of the lopinavir assay validation were between 95.0% and 96.4%, and between 96.2% and 99.1%. The coefficient of variation (%CV) was less than 3.9%.

### Pharmacokinetic analysis

The main pharmacokinetic measures for rifabutin, d-RBT and lopinavir were derived by non-compartmental analysis using Stata (StataCorp. 2009. *Stata Statistical Software: Release 11*. College Station, TX: StataCorp LP). The peak concentration (C_max_), and time to C_max_ (T_max_) were obtained directly from concentration-time profiles. Drug concentrations at the end of a dosing interval are reported as C_min_ and pre-dose concentrations as C_0._ The steady-state AUC from time 0 h to the last quantifiable sample at 24 h (AUC_0–24_) or 48 h (AUC_0–48_) for rifabutin and 12 h (AUC_0–12_) for LPV/r were calculated by the linear trapezoidal method. The apparent total oral clearance of rifabutin from plasma at steady state (CL/F) was calculated by dose/AUC.

### Statistics

For statistical analysis, the AUC_0–24_ was log-transformed. A linear mixed model with two doses (high and low), day (2 and 3), the sequence of the doses, log AUC_0–24_ and id nested within sequence was used. As there was no significant effect of sequencing of the doses (whether the patients received the tiw dose before the daily dose of rifabutin or vice versa), the two corresponding doses from each arm were pooled for further analysis. A paired t-test was used to compare the AUC_0–24_ for the 150 mg daily dose with that for 150 mg tiw and 300 mg daily doses. An AUC_0–48_ was derived for the 150 mg daily and 300 mg daily doses by doubling the AUC_0–24_. This was compared with the AUC_0–48_ for the 150 mg tiw dose using a paired t-test. The dosing interval is 48 hours for the tiw dose for 2 of 3 doses.

To calculate geometric mean ratios (GMR) for AUC, log means and 90% confidence limits were back transformed and presented in their original units as geometric means. Geometric mean ratios for the AUC of rifabutin: 150 mg daily with LPV/r / 300 mg daily, and 150 mg tiw with LPV/300 mg daily, respectively, were computed. A P-value < 0.05 was considered significant. Inter-patient variability was measured by co-efficient of variation (%CV) that was calculated as {100 X (e (var est) -1)^1/2^}. Baseline and final log viral loads and CD4^+^ counts were compared using paired t-tests.

## Results

### Patient demographics

Sixteen patients received LPV/r therapy with rifabutin. Two patients were prematurely withdrawn from the study and were therefore not evaluable for pharmacokinetic analysis, one due to uveitis and another due to non-compliance with trial medication. All patients were Black South Africans and (64%) were male. All patients had not previously received any antiretroviral therapy. The evaluable subjects’ mean (SD) age was 31.5 (5.8) years, weight was 59.9 (9.7) kg, height was 160 (7.7) cm, BMI was 23.3 (2.6), Karnofsky score Q was 100% (100) and CD4^+^ lymphocyte count was 150.9 (12.1) cells/mm^3^.

### Rifabutin and 25-O-desacetylrifabutin pharmacokinetic analysis

**Figure 2:**
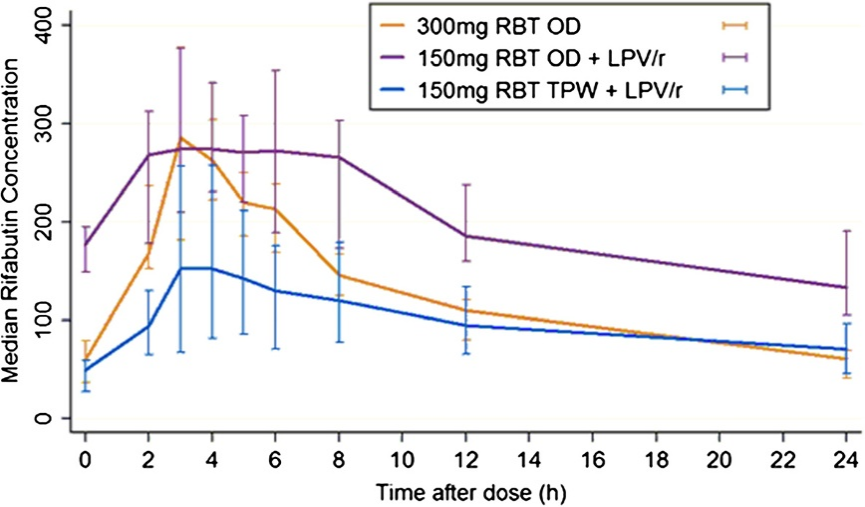
**Rifabutin median concentration-time profiles.** Median rifabutin (RBT) concentrations for the three pharmacokinetic evaluations in 14 patients. The orange line corresponds to the dosing of RBT at 300 mg without ART; the blue line to RBT dosing at 150 mg tiw with ART and the purple line to dosing with 150 mg of RBT daily with ART. The bars represent interquartile range (IQR).

**Figure 3:**
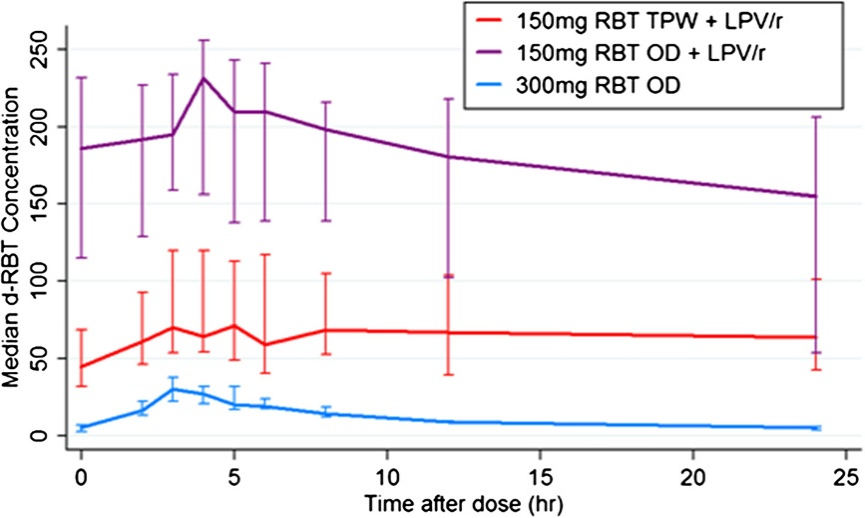
**25-O-desacetylrifabutin median concentration-time profiles.** Median 25-O-desacetylrifabutin (d-RBT) concentrations for the three pharmacokinetic evaluations in 14 patients. The bars represent interquartile range (IQR). The blue line corresponds to the dosing of RBT at 300 mg without ART; the red line to RBT dosing at 150 mg tiw with LPV/r based ART and the purple line to dosing with 150 mg of RBT daily.

The main pharmacokinetic parameters for rifabutin and d-RBT are summarized in Table 1 and shown graphically in Figures 2 and 3. The AUC_0–24_ of rifabutin 150 mg daily with LPV/r was significantly higher when compared to the AUC_0–24_ of rifabutin 300 mg daily in the absence of LPV/r (p = 0.004). In contrast, the AUC_0–48_ of rifabutin 150 mg tiw with LPV/r was significantly lower than the AUC_0–48_ of rifabutin 300 mg daily (p = 0.0001). These differences were large as demonstrated by the GMR (Table 2). The GMR (90% CI) for AUC_0–48_ was 0.6 (0.5-0.7) and 0.5 (0.4-0.6) for rifabutin 150 mg tiw compared with rifabutin 300 mg. For the comparison of the 150 mg daily dose of rifabutin with the 300 mg dose the GMR of the AUC_0–24_ was 1.6 (1.4-1.9). Wide inter-patient variability was observed in rifabutin AUC for all three doses (Table 2). The %CV was 24% for the 300 mg dose, 46% for rifabutin 150 mg daily plus LPV/r and 52% for rifabutin 150 mg tiw plus LPV/r.

**Table 1:** **Pharmacokinetic parameters for rifabutin and 25-**O***-*** **desacetylrifabutin for each study treatment**

Treatment period	Rifabutin 300 mg	Rifabutin 150 mg tiw plus LPV/r	Rifabutin 150 mg daily plus LPV/r
**Rifabutin (n = 14)**			
AUC_0–24_ (ng.h/mL)	3052.9 (2650.2-3431.5)	2307.5 (1767.5-3884.0)	4766.0 (3950.5-6099.5)
AUC_0–48_ (ng.h/mL)	6105.8 (5300.4-6863.0)*	3402.1 (2809.2-6092.0)	9532.0 (2238.2-22425.4)*
C_max_ (ng/mL)	291.5 (250.0-377.0)	167.5 (87.8-294.0)	311.0 (258.0-376.0)
T_max_ (h)	3.0 (3.0-4.0)	3.5 (3.0-5.0)	3.0 (3.0-4.0)
C_0_ (ng/mL)	59.0 (36.4-78.6)	49.1 (27.7-58.9)	176.5 (149.0-195.0)
C_min_ 24 h (ng/mL)	60.7 (40.6-68.8)	70.7 (45.7-96.6)	133.0 (105.0-191.0)
C_min_ 48 h (ng/mL)	-	37.0 (26.6-70.0)	-
CL/F (L/h)	98.3 (87.4-113.2)	65.2 (38.6-85.0)	31.5 (25.0-38.0)
AUC_0–24_ (ng.h/mL) (Rifabutin + Metabolite)	3402.3 (2900.3-3717.2)	3937.2 (2424.6-6772.7)	8753.0 (7771.7-11 505.0)
**d-RBT (n = 14)**			
AUC_0–24_ (ng.h/mL)	273.3 (235.7-344.1)	1565.5 (1105.5-2567.3)	4118.0 (2678.2-5405.5)
AUC_0–48_ (ng.h/mL)	546.6 (471.4-688.2)^*^	2318.2 (1722.9-4685.9)	8236.0 (5356.4-10811.0)^*^
C_max_ (ng/mL)	32.5 (25.2-37.7)	77.2 (58.6-128)	236.5 (159.0-274.0)
T_max_ (h)	3.0 (3.0-4.0)	5.0 (4.0-6.0)	4.0 (3.0-3.0)
C_0_ (ng/mL)	5.1 (2.7-6.6)	44.6 (31.7-68.9)	186.0 (115.0-232.0)
C_min_ 24 h (ng/mL)	5.0 (3.4 -5.8	63.9 (42.7-101.0)	155.0 (53.6-206.0)
C_min_ 48 h (ng/mL)	-	35.4 (27.7-81.0)	-

Parameters are median values (interquartile range).

*calculated by 2X AUC_0–24_.

RBT = rifabutin.

d-RBT =25-O-desacetylrifabutin.

LPV/r = lopinavir/ritonavir based ART.

tiw = three times per week.

AUC = area under the curve.

C_max_ = maximum concentration in plasma.

T_max_ = time at which maximum plasma attained.

CL/F = clearance.

C_0_ = pre-dose concentration.

C_min_ = trough concentration.

**Table 2:** **Geometric mean ratios of rifabutin and 25-O-desacetylrifabutin parameters with and without antiretroviral therapy**

	GMR (90% CI)		
	RBT 150 mg tiw with RBT 300 mg daily	RBT 150 mg tiw with RBT 150 mg daily	RBT 150 mg daily with RBT 300 mg daily
AUC_0–24_	0.8 (0.7 – 0.9)	0.4 (0.5 – 0.5)	1.6 (1.4 – 1.9)
AUC_0–48_*	0.6 (0.5 – 0.7)	0.4 (0.4 – 0.4)	n/a
C_max_(ng/mL)	0.5 (0.4 – 0.6)	0.5 (0.5 – 0.6)	1.0 (0.9 – 1.0)
C_0_(ng/mL)	0.7 (0.5 – 0.9)	0.2 (0.1 – 0.3)	3.4 (3.7 – 3.1)
C_min_24h(ng/mL)	1.2 (1.0 – 1.4)	0.5 (0.4 – 0.5)	2.7 (2.2 – 3.2)

*calculated by 2 times the AUC_0–24_ for the RBT 300 mg daily and RBT 150 mg daily arms.

n/a – Not applicable.

GMR – Geometric mean ratio.

90% Cl – 90% confidence interval.

RBT 150 mg tiw – rifabutin dose of 150 mg three times per week (tiw) in combination with lopinavir/ritonavir based ART and isoniazid.

RBT 150 mg daily – rifabutin dose of 150 mg daily in combination with lopinavir/ritonavir based ART and isoniazid.

RBT 300 mg daily – rifabutin dose of 300 mg daily in combination with isoniazid.

The C_max_ of rifabutin 150 mg tiw with LPV/r was also significantly lower when compared to the 150 mg daily dose with LPV/r (P = 0.01) and the 300 mg daily dose without LPV/r (P = 0.01). The GMR (90% CI) for Cmax was 0.5 (0.4-0.6) for RBT 150 mg tiw compared with RBT 300 mg and 0.5 (0.5-0.6) for RBT 150 mg tiw compared with 150 mg daily. The median C_min_ for rifabutin 300 mg was 60.7 ng/mL (IQR, 40.6-68.8 ng/mL). The C_min_ values increased in the presence of LPV/r with daily dosing of rifabutin but dropped significantly with the tiw dose. Rifabutin clearance was significantly reduced in the presence of LPV/r (p = 0.001 for daily and p = 0.002 tiw rifabutin dosing) compared to 300 mg rifabutin given alone.

Without lopinavir, d-RBT concentrations were 11% of the parent drug. Plasma d-RBT concentrations increased 5-fold with tiw rifabutin dosing and 15-fold with daily doses of rifabutin (Figure 3). The total antimicrobial moiety (combined AUC_0–24_ of rifabutin and metabolite) for rifabutin at 150 mg tiw with ART was 1.2 times greater than for 300 mg rifabutin and 2.6 times less than for 150 mg daily with ART.

### Lopinavir pharmacokinetic analysis

**Figure 4:**
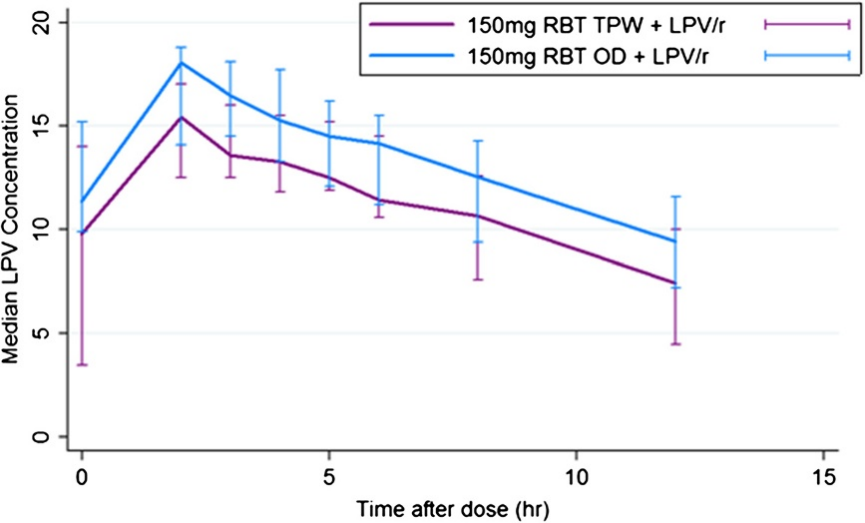
**Median concentration-time profile of boosted lopinavir administered with two different doses of rifabutin.** The median lopinavir (LPV) concentrations for 14 patients administered 2 different concentrations of rifabutin (RBT). Blue line corresponds to a RBT dose of 150 mg daily and the purple line to 150 mg tiw. LPV/r corresponds to boosted lopinavir. The bars represent interquartile range (IQR).

Lopinavir pharmacokinetic measures are shown in Table 3 and Figure 4. Median lopinavir trough (C_0_) concentrations were above the recommended lower limit for ART-naïve patients of 1 μg/ [23]. Although there was a trend to higher lopinavir concentrations with the once daily dosing of rifabutin, the differences in AUC_0–12_ and C_max_ between the two doses were not significant. Double peaks were observed in the individual lopinavir concentration-time profiles with both doses of rifabutin.

**Table 3:** **Pharmacokinetic parameters for lopinavir for each study treatment**

	Median (Interquartile range)	
Parameter	RBT 150 mg tiw plus LVP/r	RBT 150 mg daily plus LPV/r
AUC_0–12_ (μg.h/mL)	139.5 (103.8-163.9)	160.1 (129.1-181.9)
C_max_ (ng/mL)	15.8 (12.9-17.1)	18.1 (14.5-19.6)
T_max_ (h)	2.0 (2.0-3.0)	2.0 (2.0-3.0)
C_0_(μg/mL)	9.8 (3.5-14.0)	11.4 (9.9-15.2)
C_min_ (μg/mL)	7.4 (4.5-10.0)	9.4 (7.2-11.6)

RBT = rifabutin.

LPV/r = lopinavir/ritonavir.

tiw = three times per week.

AUC = area under the curve.

C_max_ = maximum concentration in plasma.

T_max_ = time at which maximum plasma attained.

C_0_ = pre-dose concentration.

C_min_ = trough concentration.

### Response to TB/HIV treatment

Three patients were culture positive after two months of tuberculosis therapy and none culture positive at the end of therapy. The mean final CD4^+^ count at the end of tuberculosis therapy was 253.8 (42.4) cells/mm^3^, and significantly higher (p = 0.03) than baseline. The mean (SD) viral load dropped significantly (p < 0.001) by 2.7 log10 copies and 8 patients had viral loads < 500 copies/ml.

### Adverse events

Adverse events (AE) were analyzed for all sixteen patients. Rifabutin was well tolerated at all doses and there was only one withdrawal because of an adverse event (uveitis). There were two serious adverse events (bacterial meningitis and pyelonephritis), both considered unrelated to rifabutin by the study team. Grade 2 uveitis occurred in one patient after 1 month of rifabutin, coinciding with the start of ART, and resolved with no sequelae after withdrawal of medication. Her AUC_0–24_ and C_max_ values for rifabutin were within the interquartile range. The commonest laboratory AE was neutropenia. Grade 3 neutropenia occurred on 7 occasions in 5 patients (Table 4). There were 2 grade 3 elevations in transaminases and amylase. There were no grade 4 laboratory events.

**Table 4:** **Timing of selected grade 3 laboratory adverse events**

Subject	AE Grade	AE	RBT dose at time of AE	Arm	Days on RBT
148	3	AST increased	150 mg daily	Low-High	106
148	3	Amylase increased	150 mg daily	Low-High	113
171	3	Neutropenia	300 mg daily	Low-High	29
182	3	Neutropenia	300 mg daily	Low-High	27
204	3	Neutropenia	150 mg daily	High-Low	57
204	3	Neutropenia	150 mg tiw	High-Low	112
242	3	Neutropenia	300 mg daily	High-Low	28
242	3	Neutropenia	150 mg daily	High-Low	53
242	3	AST increased	150 mg tiw	High-Low	96
242	3	Amylase increased	150 mg tiw	High-Low	119
250	3	Neutropenia	150 mg daily	Low-High	77

AE – adverse event.

RBT – rifabutin.

ARM – refers to the sequencing order of the rifabutin dosing in combination with ART.

tiw- three times per week.

## Discussion

The results of this study show that there are substantial differences in the AUC_0–24_ of rifabutin obtained with the two different dosing regimens in combination with LPV/r. The daily dose of 150 mg resulted in a more than two fold increase in the AUC_0–24_ when compared to the three times a week dose. The difference between the two doses was even greater when comparing the AUC_0–48_. There is a lack of conventional efficacy data in support of a particular dose or target pharmacokinetic parameter for rifabutin [24], but there is convincing evidence that intermittent rifamycin therapy is associated with tuberculosis relapse and rifamycin resistance, especially in subjects with low CD4 counts [17, 18, 25–27]. In TBTC study [18], TB-HIV co-infected patients who had AUC_0–24_ < 4.5 μg.h/mL were at higher risk of ARR. Of the patients who relapsed or failed therapy 83% developed ARR as opposed to 33% who had AUC’s above this threshold value. In this study, 71% patients on rifabutin 150 mg daily had AUC_0–24_ values >4.5 μg.h/mL compared to 14% on rifabutin tiw dosing. Similarly the C_min_ values of rifabutin 48 hours after dosing tiw are significantly lower than the C_min_ values for 300 mg daily and 150 mg daily. On the basis of prevention of resistance our data support the guidelines that recommend a dose of rifabutin 150 mg daily in combination with protease inhibitors [28].

The AUC_0–24_ and C_max_ of d-RBT were significantly increased in the presence of LPV/r in keeping with previous treatment [29, 30] and healthy volunteer studies [11, 31, 32]. There were respective increases in exposure to the metabolite of approximately 5- and 15-fold when rifabutin 150 mg was given tiw or daily with LPV/r and are probably due to the presence of ritonavir. Ritonavir is a potent inhibitor of CYP3A4, which metabolizes d-RBT. d-RBT is known to have significant anti-mycobacterial activity and could contribute to the regimen efficacy [19]. These elevations of the d-RBT metabolite could led to an increase in adverse drug reactions. We were unable to show a significant association between plasma rifabutin and d-RBT concentration and adverse events such as neutropenia or elevated transaminases however the numbers of patients are few and larger studies are required to establish the safety of the rifabutin 150 mg daily dose. It will also be important to investigate the interaction of RBT in combination with other antiretrovirals [33].

Although the currently recommended dose of rifabutin for the treatment of pulmonary tuberculosis is 300 mg daily there are few pharmacokinetic data from HIV infected African tuberculosis patients treated with this dose. The median rifabutin AUC_0–24_ and C_max_ values from this study are comparable to previous studies of rifabutin 300mg daily in HIV-infected patients [15, 34–36]. The pharmacodynamic-pharmacokinetic (PKPD) relationship for rifabutin has not been comprehensively studied so it is not clear how the reduced AUC of the 150 mg tiw dose relative to the 300 mg dose without ART reported here would impact on tuberculosis treatment outcomes. It is still uncertain if C_max_ or AUC is the critical pharmacodynamic measure for rifamycins. Mitchison [37] and others reported the C_max_/MIC to be the best PKPD measure whereas subsequent murine and hollow fibre models [38, 39], and early bactericidal activity studies in humans [40] found that the AUC_0–24_/MIC ratio was a superior parameter.

Previous studies of the pharmacokinetic interaction of rifabutin with LPV/r in HIV infected individuals have mostly been small case series or involved an adaptive design in which only selected patients were exposed to the higher dose of rifabutin [15, 16, 41]. One previous study has reported on the rifabutin pharmacokinetics in combination with LPV/r (Aluvia) but in patients initiating rifabutin at the start of therapy [42]. This study was conducted in Vietnam in a different population group but also reported that the 150 mg tiw dose was potentially sub therapeutic when compared to the 150 mg daily dose or the 300 mg dose without ART. Similar to that study we adopted a cross-over design in which all patients received 3 full pharmacokinetic assessments with rifabutin alone and at two different rifabutin doses in combination with ART allowing us to formally compare the different dosing strategies and reduce intra-patient variability. However there are limitations to our study. Although patients received rifabutin for a total of 18 weeks our numbers are small so it is necessary to be cautious in drawing definitive conclusions about the safety of rifabutin at the higher dose. We included immune-suppressed HIV positive patient but not those with CD4 count less than 50, and it is conceivable that the pharmacokinetics and tolerability of rifabutin may be different in the most highly immune-suppressed group of patients.

Although the higher dose (150 mg daily) used in this study resulted in rifabutin levels that reduce the risk of resistance it is important to emphasis that the approximately 15 fold increase in the d-RBT metabolite might result in an increase in adverse events. Neutropenia and uveitis have previously been identified as severe adverse events associated with the co-administration of rifabutin with a CYP3A4 inhibitor [43–47]. A recent paediatric clinical trial of rifabutin in combination with LPV/r was stopped due to a high frequency of grade 4 neutropenia [14]. An advantage of this study is that patients remained on study doses for sixteen weeks allowing a safety evaluation to be made over a longer duration. In this study the combinations of rifabutin and LPV/r were generally well tolerated with no grade 4 toxicities apart from 2 clinical serious adverse events reported by the study investigators as unrelated to rifabutin. Neutropenia was a common adverse event and has been reported predominantly in previous studies on healthy volunteers, but there were no grade 4 cases. Although there was a significant fall in the neutrophil count during the course of the trial most of this decline occurred in the first few weeks of rifabutin therapy prior to the initiation of ART and we did not find a significant association between neutropenia and plasma rifabutin concentrations. Neutropenia is also the most common side effect of cotrimoxazole therapy in HIV- infected patients [48] and all patients in the present study were prescribed cotrimoxazole 960 mg daily as prophylaxis. The majority of the patients started cotrimoxazole at enrollment and this would have contributed to the declining neutrophil count seen in this study. Uveitis occurred only once in a participant who had been taking 300 mg rifabutin and was not associated with high serum concentrations of drug suggesting it could have been HIV related rather than drug related. However the number of patients are small in this study so the safety of the 150 mg daily rifabutin dose needs to be established in a larger cohort.

The activity of protease inhibitors is influenced by their concentrations in plasma [49] therefore the pharmacokinetics of LPV/r were evaluated in the presence of rifabutin. The median LPV/r AUC_0–12_, C_max_, C_0_ and C_12_ obtained in this study when LPV/r was administered with two different doses of rifabutin are consistent with historical control data [50]. Secondary peaks were observed in the time-concentration profiles of LPV/r, usually within 4 hours of drug ingestion, similar to patterns observed in other studies. In both dosing arms, median LPV/r trough (C_0_) concentrations at steady state were above the recommended lower limit for ART-naïve patients of 1 μg/mL [23] and therapeutic LPV/r trough (C_0_) and C_min_ (C_12_) concentrations were achieved in all participants with both doses of rifabutin.

## Conclusions

In conclusion this study supports the recent change to some guidelines for the dosing of rifabutin in combination with LPV/r [28]. The high proportion of participants on the 150 mg tiw arm who failed to achieve rifabutin concentrations that prevented the emergence of drug resistance when the drug is dosed twice weekly is concerning. Although escalating the rifabutin dose after therapeutic drug monitoring is a viable option in resource rich settings, it is impractical in many regions of the world where HIV and TB are endemic. Our study was too small to address all concerns about the toxicity of the higher dose rifabutin with LPV/r, most notably the decrease in neutrophil count, which requires further evaluation.
